# Benchmark Force Fields for the Molecular Dynamic Simulation of G-Quadruplexes

**DOI:** 10.3390/molecules26175379

**Published:** 2021-09-04

**Authors:** Na Li, Ya Gao, Feng Qiu, Tong Zhu

**Affiliations:** 1 Shanghai Engineering Research Center of Molecular Therapeutics & New Drug Development, School of Chemistry and Molecular Engineering, East China Normal University, Shanghai 200241, China; naliz@foxmail.com; 2 School of Mathematics, Physics and Statistics, Shanghai University of Engineering Science, Shanghai 201620, China; 3 Institute of Artificial Intelligence on Education, Shanghai Normal University, Shanghai 200234, China; 4 NYU-ECNU Center for Computational Chemistry, New York University Shanghai, Shanghai 200062, China; 5 Shandong Key Laboratory of Biophysics, Institute of Biophysics, Dezhou University, Dezhou 253023, China

**Keywords:** G-Quadruplex, molecular dynamics simulation, force field, polarization effect

## Abstract

G-quadruplexes have drawn widespread attention for serving as a potential anti-cancer target and their application in material science. Molecular dynamics (MD) simulation is the key theoretical tool in the study of GQ’s structure-function relationship. In this article, we systematically benchmarked the five force fields of parmbsc0, parmbsc1, OL15, AMOEBA, and Drude2017 on the MD simulation of G-quadruplex from four aspects: structural stability, central ion channel stability, description of Hoogsteen hydrogen bond network, and description of the main chain dihedral angle. The results show that the overall performance of the Drude force field is the best. Although there may be a certain over-polarization effect, it is still the best choice for the MD simulation of G-quadruplexes.

## 1. Introduction

G-Quadruplexes (GQs) [1] are folded by guanine-rich nucleic acid sequences. Four guanines linked by Hoogsteen hydrogen bonds can constitute a planar square called a tetrad. Guanine tetrads can stack together driven by pi-pi interaction and the dynamics of the sugar-phosphate backbone. Monovalent cation is another main stabilization factor of GQs, which can coordinate with the carbonyl oxygen atoms in the nucleobases and thus reduce the repulsion between these negative charged groups [2,3,4]. Previous studies have found that there are nearly 400,000 G-quadruplex sequences in the human genome, and about 40% of the human genome promoter region contains more than one G-quadruplex sequence [5,6], which are one of the main factors for maintaining chromosome stability. GQs are involved in the process of gene replication, transcription, and translation [7,8,9], and are inseparable from the formation and development of cancer [10,11,12,13,14,15,16]. In addition, GQs are widely used in the field of material science and medical treatment owing to their unique properties of polymorphism and ionic affinity [17,18]. In order to make better use of GQs in these fields, we must deeply understand the relationship between their structures and functions. Since the first discovery of GQ in 1960s [19], considerable endeavors have been made for this purpose [20]. In the experimental community, hundreds of high-resolution three-dimensional structures of GQs have been determined by nuclear magnetic resonance (NMR) and X-ray crystallography. In the theoretical computation community, molecular dynamics (MD) simulation and quantum methods [21] give essential insights to GQs. Interestingly, many works have found that the structures of GQs are highly sensitive to the sequences, base orientations, metal ions, and environmental PH [22,23]. Therefore, it is necessary to study the dynamic properties of GQ, and this is exactly what theoretical simulations, especially MD simulation methods [24,25], excel at.

Noteworthily, reliable results of MD simulation highly depend on accurate force fields. In the past three decades, many force fields have been developed for canonical DNA or RNA. In 1995, Kollman and co-workers proposed the parm94 [26] force field and then updated it to parm98/99 [27,28] in 1998. Whereas in 2007, Pérez et al. proposed the parmbsc0 force field [29] by refining the α/γ torsional terms of parm99. Similar strategies were used in the development of the OL15 [30] and parmbsc1 [31] force fields. CHARMM27 and its updates such as CHARMM36 were also proposed and support the simulation of nucleic acids [32,33]. Although these force fields are often used in the MD simulation of GQs [34,35,36,37], the simulated results are not always satisfactory. For example, bifurcated hydrogen bonds [34] are often observed in these simulations but they are not discovered in the experimental structures. In addition, it has been found that when the classical pair-additive force field is used, the metal ions in the ion channel of GQs are usually not stable [38,39]. There are two main reasons for the inaccuracy of classical force fields. First, their parameters are not optimized specifically for GQs. Šponer and co-workers have shown that after optimization of water and ion parameters for GQ, the performance of the traditional force field can be obviously improved [40,41]. The second and more critical reason is that classical force fields lack explicit inclusion of important quantum effects, especially electronic polarization. Classical force fields often use fixed-point charge model to calculate the electrostatic interactions in the system, which cannot reflect the change of the electrostatic environment of the system with the structure. However, the strong polarity of nucleotides, the complicated Hoogsteen hydrogen bonds, and the participation of metal cations make the polarization effects play a more important role in the GQs than in other biomolecules. In their previous study, Song et al. developed the polarized nucleic acid-specific charge (PNC) model [38]. By using this model, they obtained a significantly improved structural description of GQ compared with Amber ff10 force field. Recently, Lemkul and co-workers showed that the Drude 2017 polarizable force field outperforms the classical CHARMM36 force field in the simulation of several GQs [39,42,43,44].

Although there are many force fields available for the MD simulation of GQ, currently none of them are widely accepted. In this work, a systematic test and evaluation of possible GQ force fields was conducted. Five force fields, parmbsc0, parmbsc1, OL15, Drude2017 and AMOEBANUC17 [45] were selected, and their performance on two DNA GQs containing K^+^ and Na^+^ ions was benchmarked. The article is organized as follows: Section 2 presents the results, Section 3 lists the calculation details and a brief discussion occurs in Section 4.

## 2. Results and Discussion

**Figure 1:**
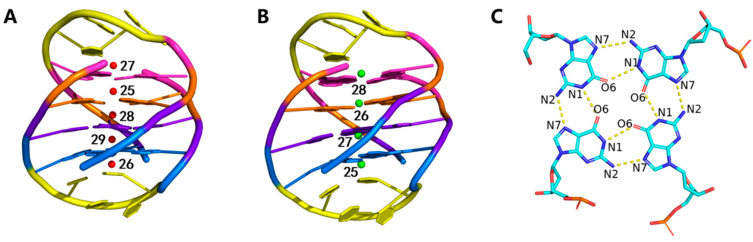
Two DNA GQs used in this study. They share the same sequence (GGGGTTTTGGGG)_2_ and 3D structure but use different metal ions as cofactors. Diagonal loops, tetrad 1, tetrad 2, tetrad 3, and tetrad 4 are colored by yellow, magenta, orange, purple, and blue, respectively. (**A**) K-GQ. K^+^ ions are colored in red. (**B**) Na-GQ. Na^+^ ions are colored in green. (**C**) Hoogsteen hydrogen bonds in tetrad 2.

There are two GQs used in the current study. The first one is a crystal structure of *Oxytricha nova* telomeric DNA GQ with the sequence of d[G_4_T_4_G_4_]_2_ [46], which use potassium ions as cofactor (PDB ID 1JRN). There are two identical GQs in this structure. To save the computational cost, only one of them was kept, which structure is shown in Figure 1A. The guanine bases in this structure adopt alternating syn-anti glycosidic bond conformations. The second one has the same sequence but uses sodium ions as cofactor (PDB ID 1JB7) [47]. It was also determined by X-ray crystallographic experiment. Water molecules in these two structures were deleted before MD simulation. We abbreviate the GQ containing potassium ions and sodium ions as K-GQ and Na-GQ, respectively. 

### 2.1. Stability of the Overall Structure

**Figure 2:**
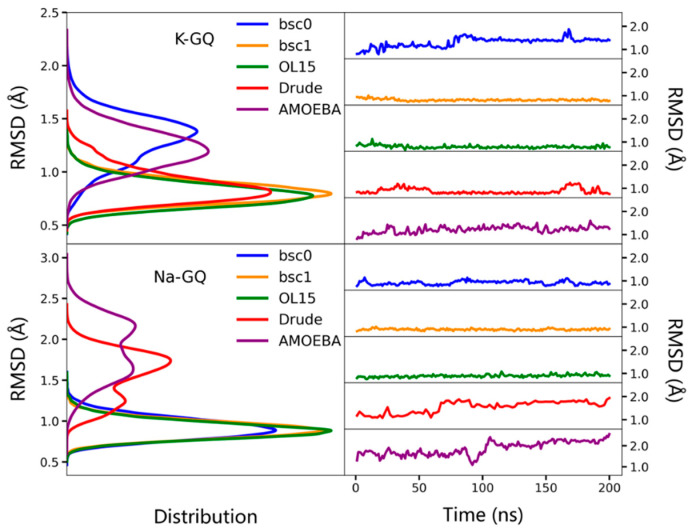
The RMSDs of backbone atoms (C3′, C4′, C5′, O3′, O5′, P) in K-GQ and Na-GQ during MD simulation with different force fields. The experimental structures were used as references. To show the results more clearly, the average RMSD value of every 100 snapshots is shown.

To depict the stability of GQs in MD simulations, the distributions of the root mean square deviation (RMSD) of backbone atoms (C3′, C4′, C5′, O3′, O5′, P) are shown in Figure 2. As can be seen, the RMSDs of K-GQ under five force fields are all smaller than 2.0 Å. The RMSDs of Drude and OL15 have the lowest value and their distributions are almost identical; while the AMOEBA gives the largest RMSD value, which indicates the obvious change of structures. For Na-GQ, the RMSD values under all force fields are slightly larger than that of K-GQ; and the GQ conformation under the AMOEBA force field again changed most. The fluctuation of the total energy of each system during MD simulation is shown in Figure S1. It can be seen that the MD simulation with the AMOEBA force field for Na-GQ has not reach equilibrium even after 200 ns. The RMSDs of another two replicas are shown in Table S1, which agree very well with the results of the first replica. 

### 2.2. Stability of Channel Ions 

**Figure 3:**
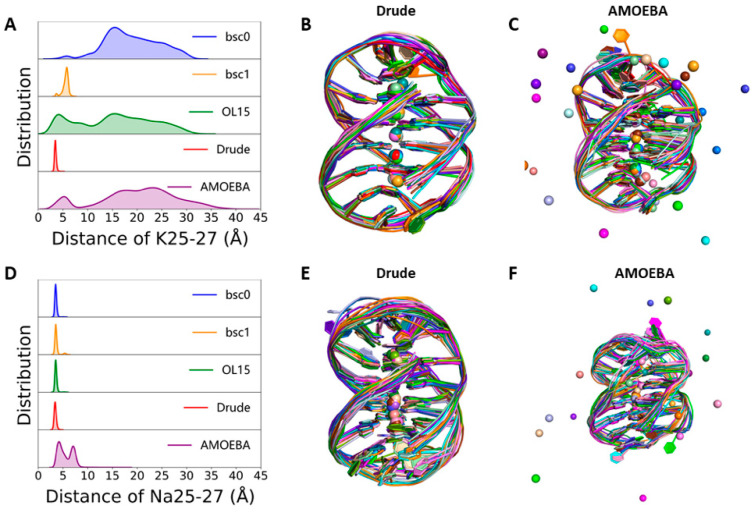
The distribution of distances between terminal ions and the comparison of experimental and simulated structures of GQ in replication 1. (**A**,**D**) are the distribution of distance between K25–K27 in K-GQ and Na25-Na27 in Na-GQ in MD simulations, respectively. (**B**–**F**) are alignments of experimental structures of K-GQ and Na-GQ with that were taken from MD simulations.

Metal ions are key factors that can stabilize the GQ structure. Many previous studies have ranked the stability of monovalent ions in GQs by experiments [48,49,50]. As the radius of K^+^ and Na^+^ are different, they occupy different positions in the GQ stem [43,51]. K^+^ ions are mainly located between two tetrads, while Na^+^ ions are usually in the plane of tetrads (Figure 1). To check the stability of ions, we monitored the distance of two adjacent ions at the terminal of the GQ channel, respectively. As displayed in Figure 3, none of channel ions escaped from the GQ in the MD simulations with the Drude force field. Although in one of the three parallel simulations using the Drude force field, the terminal K^+^ ions escaped, in general, Drude can better stabilize the ion channel structure (Table S2). However, in the MD simulations of K-GQ with other four force fields, at least one terminal ion went into the solvent within 5 ns (Figure S2). Figure 3B,C show the alignment of the experimental structure with 20 snapshots from MD trajectories with Drude and AMOEBA force fields, respectively. As can be seen, the position of metal ions was well preserved during the simulation with the Drude force field. By contrast, the terminal ions went into the solvent during the simulation with the AMOEBA force field, and the GQ conformation was slightly distorted without surprise. In the simulation using the OL15 force field (Figure S4), after K27 and K26 escaped into the solvent, K29 gradually moved to occupy the initial position of K26. Then, the K27 went back to its original position from solvent and escaped from the channel again after about 20 ns. When there were only three K^+^ ions left in the channel, some counterions approached the vicinity of two loops, moved into the loop region but finally left. After the escape of K^+^ ions, the planar structure of the G-tetrads is slightly distorted (Figure S5). Such a phenomenon was also found in simulations using the bsc1 and bsc0 force fields. In the simulation with the bsc1 force field, although the K27 did not go out of the GQ channel, its distance with K25 was obviously beyond the normal range [51]. When the AMOEBA force field was used, the K26 and K27 went out from channel first, and K25 moved to the position of original K27, followed by K28 moving to the position of original K25. Therefore, there was no K^+^ ion left between tetrad 2 and tetrad 3. 

Different phenomena were observed for Na-GQ. In the MD simulation with all Amber force fields, none of Na^+^ ions escaped. While in the MD simulation with the AMOEBA force field, the Na^+^ ion in one terminal escaped within 2.5 ns. (Figure S3) This phenomenon was found in two of the three parallel simulations. Although the Na^+^ ion in another terminal did not escape, its distance to its neighbor ion was very large. It is worth to mention that the structures of Na-GQ and K-GQ are the same. However, the stability of ions of these two systems is obviously different (Figure S4). This phenomenon is mainly due to the force field parameters, especially the ion radius. Classical force fields may overestimate the repulsion between relatively larger K^+^ ions, which was also found in previous quantum chemistry calculations [38,52]. The distance of terminal ions in replications is shown in Table S2. When the distances of terminal channel ions are larger than 4.0 Å, their positions can fluctuate easily and finally went into solvation. Given that the simulations using AMOEBA force field are extremely slow and the ions always escape from GQ stems within 6 ns, longer simulation time of replications is unnecessary.

### 2.3. Stability of Hoogsteen Hydrogen Bonds

**Figure 4:**
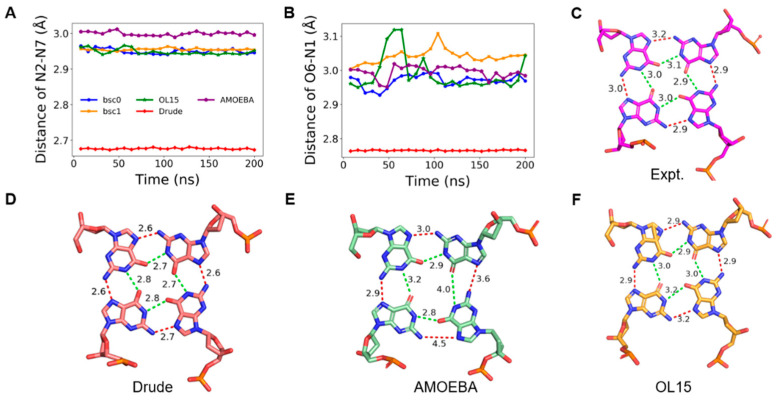
(**A**,**B**) are changes of hydrogen bond lengths in tetrad 1 during the MD simulation of K-GQ. (**C**–**F**) are typical structures of tetrad 1 in experiment, and MD simulation with Drude, AMOEBA, and OL15, respectively. (**A**,**B**) share the same figure legend.

**Figure 5:**
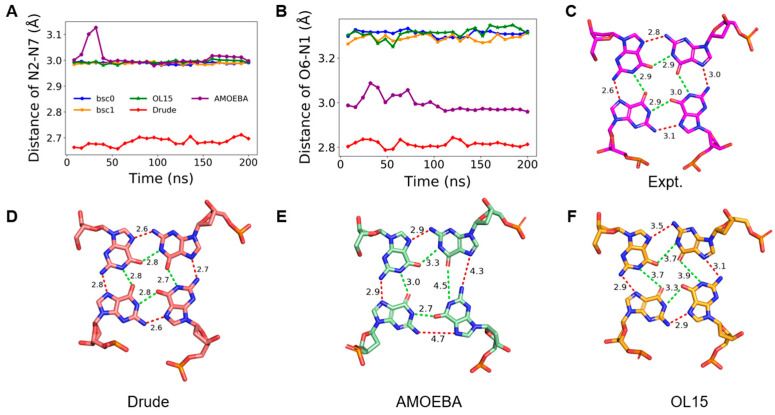
(**A**,**B**) are changes of hydrogen bond lengths in tetrad 1 during the MD simulation of Na-GQ. (**C**–**F**) are typical structures of tetrad 1 in experiment, and MD simulation with Drude, AMOEBA, and OL15, respectively. (**A**,**B**) share the same figure legend.

Adjacent guanines in a tetrad can form two types of circular Hoogsteen hydrogen bonds, which are named N1-O6 and N2-N7, respectively (Figure 1). Each tetrad contains four N1-O6 and four N2-N7 hydrogen bonds, which play a critical role in maintaining the stability of GQ structure. We inspected the distance distribution of the two types of hydrogen bonds in K-GQ and Na-GQ as presented in Figures S6 and S7. For a solid hydrogen bond, the distance between donor and acceptor should be no more than 3.5 Å. In this aspect, the performance of all force fields is acceptable, except that the distance of N2–N7 in the tetrad 4 of Na-GQ is over long after the MD simulation with the AMOEBA force field. For both GQs, the MD simulation based on the Drude force field gives the shortest hydrogen bond distance, which is about 0.1–0.3 Å shorter than the experimental value. In contrast, the donor-acceptor distances obtained from other force fields are all longer than the experimental value, and the performances of OL15, bsc0, and bsc1 are very close to each other. Typical structures of Hoogsteen hydrogen bonds in tetrad 1 from MD simulations with the experimental structures are shown in Figure 4 and Figure 5. They clearly show that the simulated Hoongsteen hydrogen bond structures by Drude and OL15 agree well with the experimental structure. However, some N2–N7 distances in the AMOEBA trajectory are longer than 4.0 Å, and the G-quartets are obviously deformed. 

**Figure 6:**
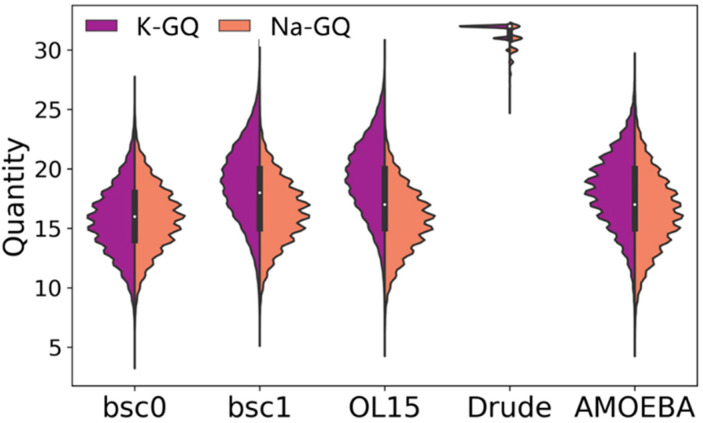
The distribution of total hydrogen bond number in the MD simulation for replication 1.

To further evaluate the performance of different force fields, we also computed the distribution of the hydrogen bond angles. As shown in Figure S8, Drude’s results are the closest to the experimental values and the distribution is narrower. Since there are eight hydrogen bonds in one tetrad, botah GQs in this study have thirty-two hydrogen bonds in total. Thus, we examined the number of hydrogen bonds in each frame and calculated their distribution (Figure 6). As excepted, the number of hydrogen bonds varies from 25 to 32 and most of them are close to 32 in Drude force field for both GQs. However, the quantity has a wider distribution, mainly from 15 to 20 in Amber and AMOEBA force fields. Figure S9 shows the occupancy of these hydrogen bonds. As can be seen, the occupation ratio is always close to 100 percent in the Drude force field, which is much higher than other force fields. 

In addition, previous study [32] has observed bifurcated N1–N7 hydrogen bonds in the MD simulation. This kind of hydrogen bond is regarded as an artifact due to the inaccuracy of the force field. Therefore, we inspected the existence of bifurcated hydrogen bonds in our MD simulations (Table S3). For the Drude force field, encouragingly, we found that none such hydrogen bonds formed throughout the MD simulation of K-GQ, whereas they existed occasionally in sodium-containing GQ. For Amber force fields, the occupation ratios of bifurcated hydrogen bonds are more than 20% in Na-GQ. For the AMOEBA force field, bifurcated hydrogen bonds are formed with a very short lifetime in both GQs. From this part, one can conclude that the formation of bifurcated hydrogen bonds can be prevented by the inclusion of polarization effect. 

Considering the hydrogen bonds interactions can be well captured by the NCI (Non-Covalent Interaction) index developed by Johnson et al. [53] and this has been widely used in previous studies [54,55,56,57,58,59], we analyzed the NCI index of G-quartets by using the Multiwfn 3.7 software [60]. The results are shown in Figure S10. It can be seen intuitively that the hydrogen bonds given by the non-polarized force fields are generally weaker than that in the experimental structure. However, the hydrogen bond is obviously stronger under the Drude force field, though we have found some repulsive interactions, which means that Drude overestimates the hydrogen bond interaction.

### 2.4. Description of Key Dihedrals

**Figure 7:**
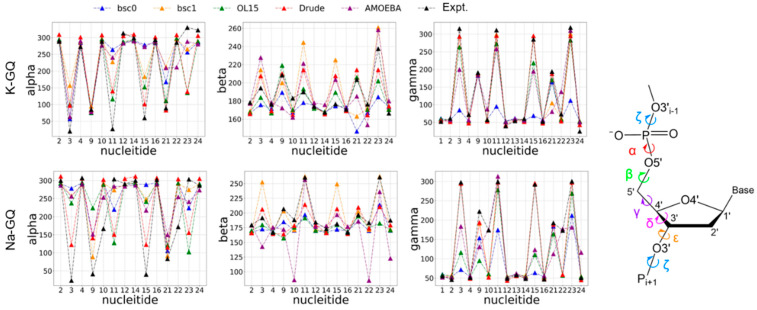
Average dihedral angle of guanine deoxyribonucleotides in replication 1 calculated from 20,000 snapshots randomly extracted from the MD trajectories of K-GQ (upper panel) and Na-GQ (bottom panel), respectively. The experimental values are also shown for comparison.

To further examine the performance of these five force fields in the description of microstructure, we compared the distribution of different dihedrals in sixteen guanine deoxyribonucleotides with experimental values (Figure 7 and Figure S11). For K-GQ, it can be seen that the simulated values based on bsc0 have the largest deviation from the experiment. As the bsc1 and OL15 were optimized for reproducing the secondary structure, their performances are better than bsc0. For the Drude force field, its performance is comparable to the OL15. For Na-GQ, there is still no force field that perfectly matches the experimental value. Overall, the performance of the Drude force field is slightly better, while the performance of other force fields is similar.

## 3. Methods

As the processes of MD simulations for both GQs are all the same, here, we take the K-GQ as example. All the MD simulations were carried out in explicit solvent with periodic boundary conditions. “Drude”, “AMOEBA”, “bsc0” and “bsc1” are the abbreviation for Drude2017, AMOEBANUC17, parmbsc0, and parmbsc1 force fields, respectively.

The tLEaP program in the AMBER18 software package [61] was used to assign the parameters of the OL15, bsc1, and bsc0 force fields on GQ. Then, the GQ was solvated in a truncated octahedral box with SPC/E water molecules. The smallest distance from any atoms of GQ to the boarder of the water box is at least 12 Å. The SPC/E water model was used according to the suggestion of the previous work [40]. Seventeen extra K^+^ ions were used to neutralize the system. The parameter proposed by Joung and Cheatham [62] were used for all K^+^ ions. Then, the structure of the system was minimized for 50,000 steps with a restraint force constant of 20 kcal/(mol·Å^2^) on the GQ and K^+^ ions in its channel, the first 25,000 steps of which are steepest descent followed by conjugate gradient. After that, the system was heated up gradually from 0 K to 300 K within 100 ps, and the restraint exerting on the same section was reduced to 5 kcal/(mol·Å^2^). Then, an unrestrained equilibration was performed for 100 ps in the NPT ensemble under 1 atm and 300 K. Finally, a 200 ns production MD was conducted with the same condition. The temperature was controlled by using a Langevin thermostat. The Particle Mesh Ewald (PME) algorithm was applied to treat the long-range electrostatic interactions [63]. A cutoff of 10 Å was used for non-bonded interactions. Hydrogen atoms were constrained by the SHAKE algorithm [64] and the time step was set to 2 fs. The Cpptraj [65] program was used to analyze MD trajectories. 

The CHARMM-GUI [66] webserver was used to build the initial system for the MD simulation with the Drude force field. The Drude Prepper in CHARMM-GUI was used to add drude particles for non-hydrogen atoms. The OpenMM 7.0 software [67] was used to perform the MD simulation. The system was minimized followed by NPT equilibration at 1 atm and 300 K through Langevin dynamics. The temperature of Drude particles was set to 1 K. Van der Waals forces were switched to zero from 10 Å to 12 Å. The friction coefficient of real atoms and Drude particles are 5 and 20, respectively. Other parameters were set to the same values as used in the MD simulation with Amber force fields except that the time step was 1 fs. For the AMOEBA force field, the Tinker-OpenMM software [68] was used to carry out the MD simulation. We performed optimization with steepest descent gradient and the RMS gradient was 0.05. In the simulation, the Andersen thermostat was switched on and a velocity Verlet integrator was adopted. The time step was set to 1 fs. Other parameters were set to same values as used in the MD simulation with Amber force fields.

For Na-GQ, the simulation protocol is almost the same as that for K-GQ, except that the counterions are Na^+^ ions. To ensure the repeatability of the results, 3 independent MD simulations have been performed for each system. The results of these 3 replicas are in good agreement. Therefore, the results of the first replica are presented in the main text, while results of replica 2 and 3 can be found in the supplementary material.

## 4. Conclusions

In this work, the performances of five force fields of parmbsc0, parmbsc1, OL15, Drude2017, and AMOEBA on the simulation of G-quadruplex were systematically benchmarked. The simulated results show that, compared with other force fields, Drude has obvious advantages in maintaining the stability of the GQ central ion channel, even though the ions escaped from channel in one replication after 80 ns. At the same time, it also performs well in maintaining the stability of the Hoogsteen hydrogen bond network. It can accurately describe the hydrogen bond angle, and it hardly leads to the production of bifurcated hydrogen bonds. However, it should be pointed out that the simulated hydrogen bond length by Drude is shorter than the experimental value, which may mean that it has a certain degree of over-polarization effect. The OL15 and bsc1 force fields can maintain the distance between the hydrogen bond acceptor and the donor, but they cannot describe the hydrogen bond angle well. In describing the dihedral angles, no force field can completely outperform others. The performances of OL15 and Drude are close, and they are in the best match with the experimental data. Although its performance on the canonical DNA system is excellent [45], the performance of the AMOEBA force field on the GQ system still has large room to be improved, which may be because it has not been specifically optimized for GQ. On the whole, the Drude2017 force field is the best choice for the MD simulation of G-quadruplex, while more precise force field development is still necessary.

## Data Availability

The data presented in this study are available in supplementary material.

## Supplementary Materials

The following are available online. Figure S1: The fluctuation of the total energy of each system during MD simulation., Figure S2: The distance of terminal ions in the K-GQ channel., Figure S3: The distance of terminal ions in the Na-GQ channel., Figure S4: The distribution of distances between terminal ions and the comparison of experimental and simulated structures of GQ. (A) and (E) are the distribution of distance between K25–K27 in K-GQ and Na25–Na27 in Na-GQ in MD simulations, respectively. (B)–(H) are alignments of experimental structures of K-GQ and Na-GQ with that are taken from MD simulations., Figure S5: The structure of G-tetrads in the experimental structure and in simulation with the OL15 force field when K+ ions escape. Figure S6: Distribution of Hoogsteen hydrogen bond distances in the K-GQ system. The line colored in cyan represents the experimental value., Figure S7: Distribution of Hoogsteen hydrogen bond distances in the Na-GQ system. The line colored in cyan represents the experimental value., Figure S8: Distribution of hydrogen bond angles during MD simulation under different force fields. Figure S9: Occupancy of 32 hydrogen bonds during MD simulation. Figure S10: Hydrogen bonds interactions of G-quartets obtained from NCI (Non-Covalent Interaction) index calculation. Figure S11: Average dihedral angle of guanine deoxyribonucleotides calculated from 2000 snapshots randomly extracted from the MD trajectories of K-GQ and Na-GQ. Table S1: RMSD of GQ backbone (C3′, C4′, C5′, O3′, O5′, P) in the MD simulation of different replicas for Na-GQ and K-GQ. Table S2: The distances of terminal channel ions in the MD simulations. Table S3: Occupation ratios of bifurcated hydrogen bonds.

## Sample Availability

Samples of the simulation details are available from the authors.
