# Supplementary material for: Benchmark Force Fields for the Molecular Dynamic Simulation of G-Quadruplexes
Source: Molecules. 2021 Sep 4;26(17):5379. doi: 10.3390/molecules26175379 (PMC8434458; doi:10.3390/molecules26175379)
Supplement: Supplementary file 1 [file molecules-26-05379-s001.zip › molecules-1343117-supplementary.pdf]

molecules

MDPI

Supporting Information

Benchmark Force Fields for the Molecular Dynamic Simulation of G-Quadruplexes

Na Li[1], Ya Gao[2*], Feng Qiu[3*] and Tong Zhu[1,4,5*]

1 Shanghai Engineering Research Center of Molecular Therapeutics & New Drug Development, School of Chemistry and Molecular Engineering, East China Normal University, Shanghai, People's Republic of China.
2 School of Mathematics, Physics and Statistics, Shanghai University of Engineering Science, Shanghai 201620, China
3 Institute of Artificial Intelligence on Education, Shanghai Normal University, Shanghai, 200234, China
4 NYU-ECNU Center for Computational Chemistry at NYU Shanghai, Shanghai, People's Republic of China
5 Shandong Key Laboratory of Biophysics, Institute of Biophysics, Dezhou University, Dezhou 253023, China

Table S1. RMSD of GQ backbone (C3', C4', C5', O3', O5', P) in the MD simulation of different replicas for Na-GQ and K-GQ. Except for the AMOEBA force field, MD simulations based on all other force fields are performed for 200ns. The AMOEBA-based replica 1 was performed for 200ns, while the replica and 3 only performed 6ns. This is because the calculation of the AMOEBA force field is extremely time-consuming and the GQ structure after 6ns simulation has already deviated far from the experimental structure.

RMSD (Å)		bsc0	bsc1	OL15	Drude	AMOEBA
Replica 1	K-GQ	1.31±0.23	0.82±0.11	0.80±0.11	0.88±0.15	1.22±0.20
	Na-GQ	0.92±0.13	0.90±0.10	0.90±0.11	1.55±0.28	1.87±0.34
Replica 2	K-GQ	1.26±0.21	0.93±0.15	1.01±0.12	1.27±0.38	0.96±0.24
	Na-GQ	0.98±0.15	0.96±0.14	0.89±0.16	1.52±0.28	1.43±0.39
Replica 3	K-GQ	1.34±0.25	0.87±0.13	0.88±0.15	0.97±0.17	0.90±0.15
	Na-GQ	1.02±0.14	0.99±0.16	0.97±0.13	1.57±0.21	1.22±0.32

* To whom correspondence should be addressed. Email: gaoya@sues.edu.cn; qxf@shnu.edu.cn; tzhu@lps.ecnu.edu.cn

Table S2. The distances of terminal channal ions in the MD simulations. Except for the AMOEBA force field, MD simulations based on all other force fields are performed for 200ns. The AMOEBA-based replica 1 was performed for 200ns, while the replica and 3 only performed 6ns. This is because the calculation of the AMOEBA force field is extremely time-consuming and the GQ structure after 6ns simulation has already deviated far from the experimental structure.

Distance (Å)		bsc0	bsc1	OL15	Drude	AMOEBA
Replica 1	K25-27	19.06±5.35	5.55±0.61	15.46±7.63	3.50±0.20	20.20±7.78
	K26-29	17.62±5.72	18.85±6.02	18.76±5.63	3.47±0.18	20.24±6.47
	Na25-27	3.57±0.23	3.69±0.47	3.58±0.25	3.45±0.24	5.59±1.48
	Na26-28	3.58±0.26	3.67±0.44	3.65±0.36	3.47±0.23	22.10±7.09
Replica 2	K25-27	18.16±6.21	16.45±5.08	15.46±7.63	3.50±0.18	4.93±0.80
	K26-29	19.21±7.24	18.17±5.62	18.76±5.63	3.53±0.21	7.11±5.69
	Na25-27	3.66±0.25	3.64±0.36	3.58±0.25	3.50±0.30	6.35±0.78
	Na26-28	3.52±0.19	3.56±0.88	3.65±0.36	3.39±0.23	16.23±8.87
Replica 3	K25-27	20.11±6.03	10.35±0.72	15.46±7.63	14.32±9.88	7.61±6.49
	K26-29	18.54±5.76	13.88±5.19	18.76±5.63	5.26±5.70	19.83±5.49
	Na25-27	3.46±0.20	3.59±0.41	3.58±0.25	3.48±0.26	4.10±0.55
	Na26-28	3.54±0.23	3.70±0.54	3.65±0.36	3.49±0.27	4.90±1.27

Table S3. Occupation ratios of bifurcated hydrogen bonds.

system	bsc0	bsc1	OL15	Drude	AMOEBA
K-GQ (%)	0.01~2.44	0.01~0.11	0.01~0.12	0.00	0.01~0.02
Na-GQ (%)	0.01~24.87	0.01~21.45	0.01~26.00	0.01~0.11	0.00~0.94

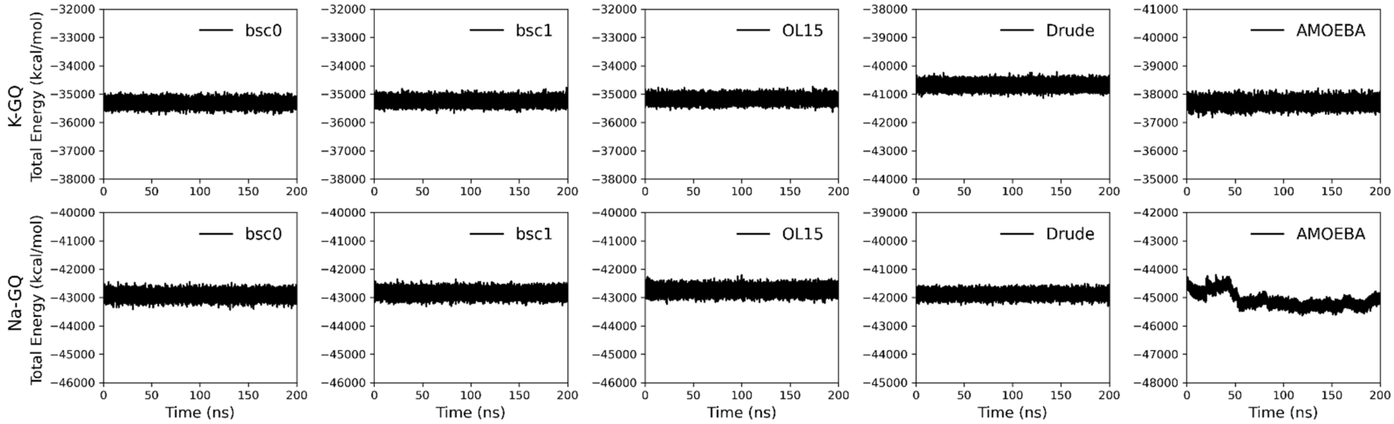

Figure S1. The fluctuation of the total energy of each system during MD simulation

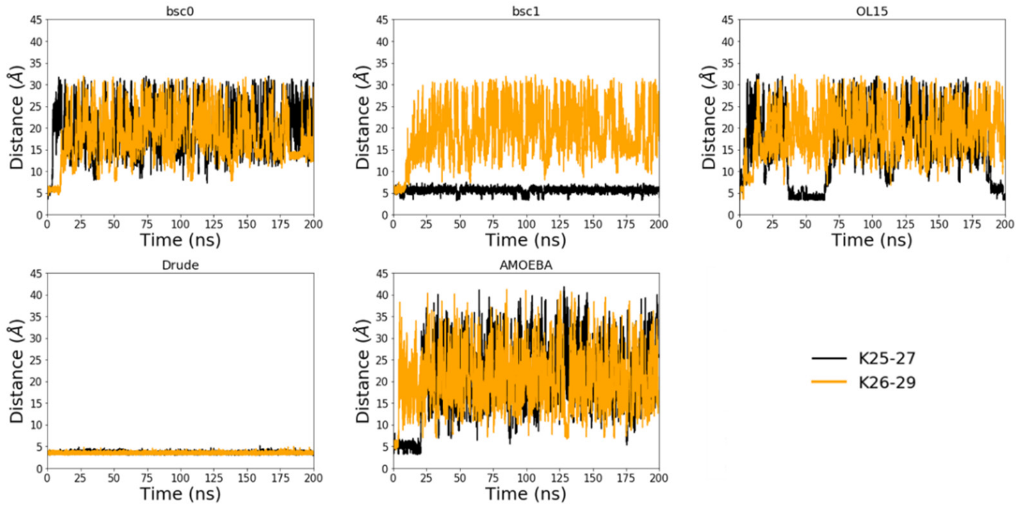

Figure S2. The distance of terminal ions in the K-GQ channel.

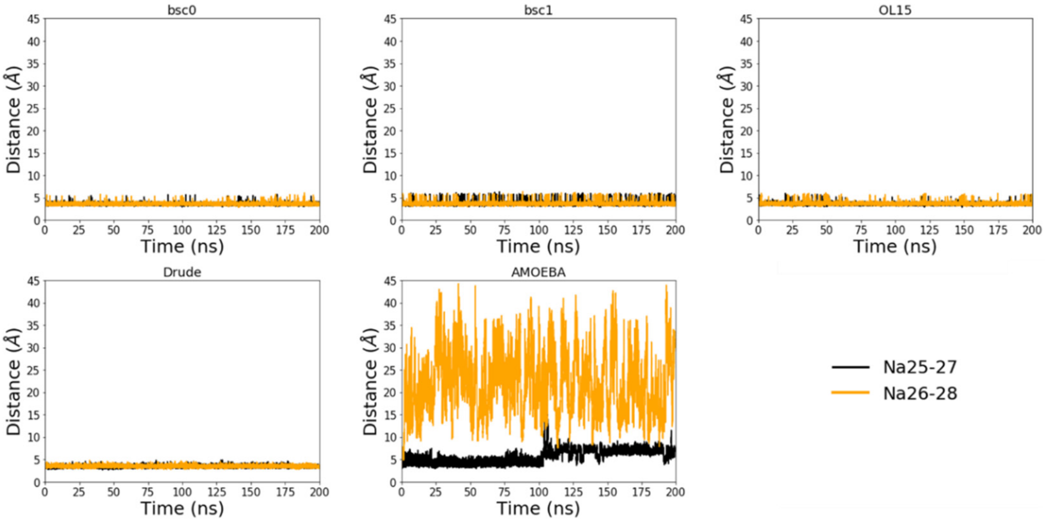

Figure S3. The distance of terminal ions in the Na-GQ channel.

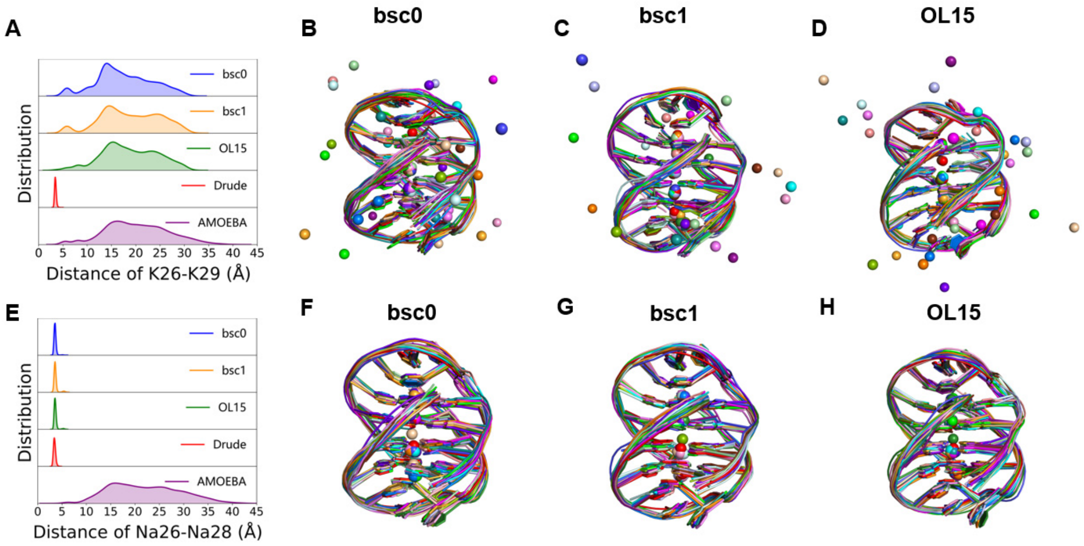

Figure S4. The distribution of distances between terminal ions and the comparison of experimental and simulated structures of GQ. (A) and (E) are the distribution of distance between K25-K27 in K-GQ and Na25-Na27 in Na-GQ in MD simulations, respectively. (B) - (D) and (F) – (H)are alignments of experimental structures of K-GQ and Na-GQ with that take from MD simulations.

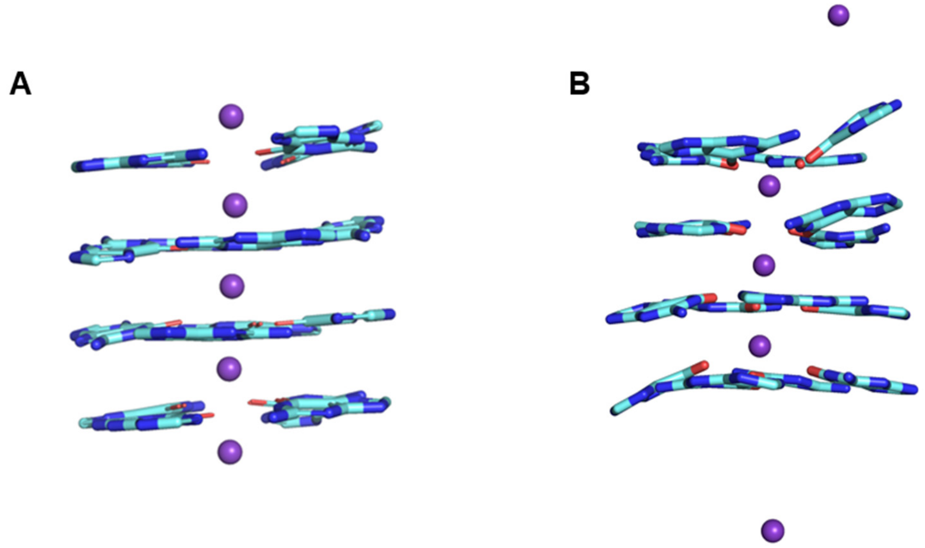

Figure S5. (A) The structure of G-tetrads in the experimental structure. (B) The structure of G-tetrads in simulation with the OL15 force field when K+ ions escape.

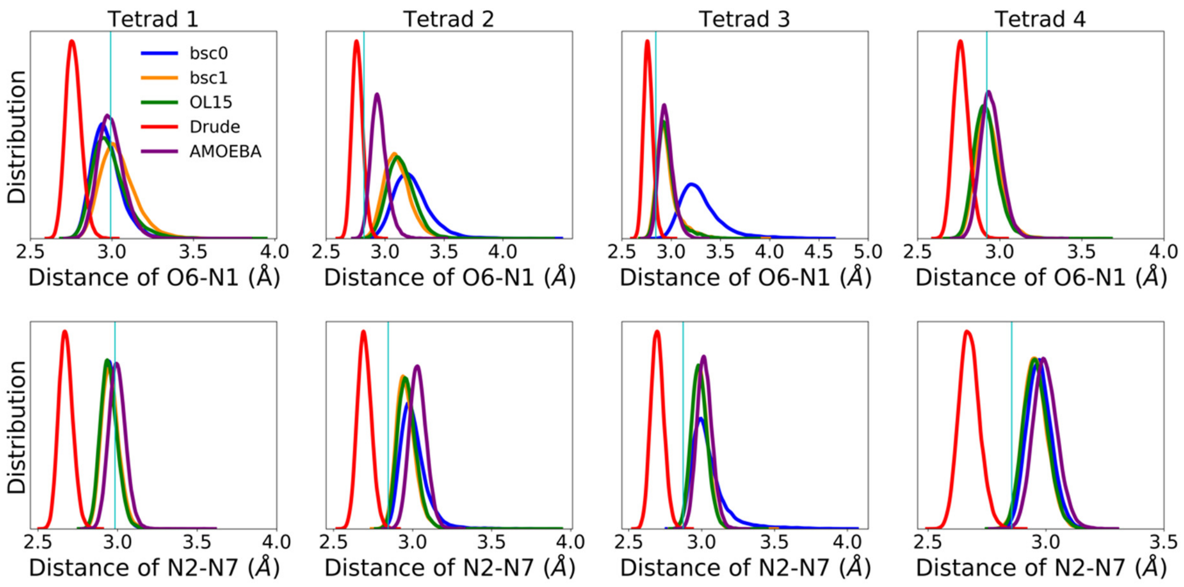

Figure S6. Distribution of Hoogsteen hydrogen bond distances in the K-GQ system. The line colored in cyan represents the experimental value.

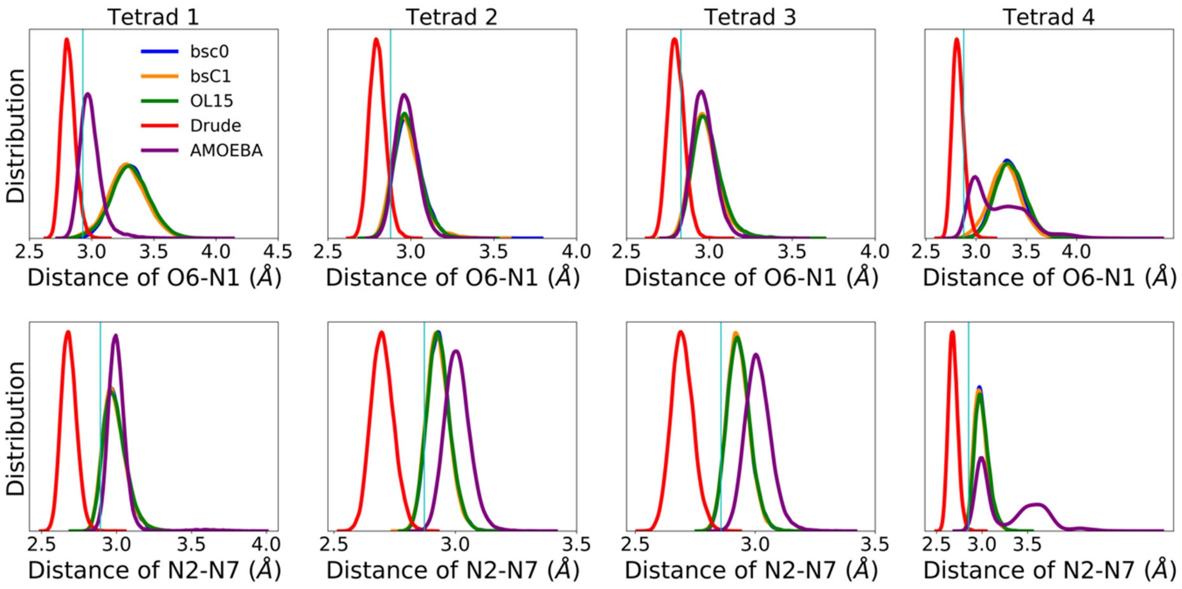

Figure S7. Distribution of Hoogsteen hydrogen bond distances in the Na-GQ system. The line colored in cyan represents the experimental value.

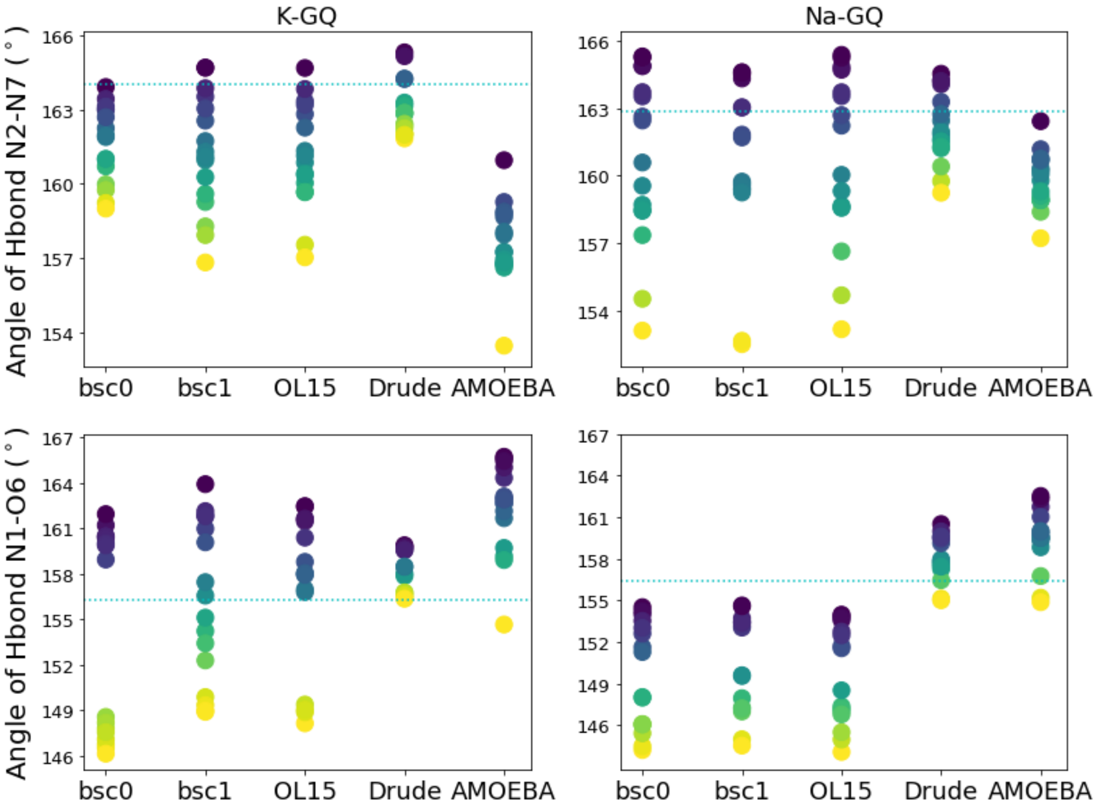

Figure S8. Distribution of hydrogen bond angles during MD simulation under different force fields. Each circle in the same column represents a hydrogen bond between different guanines in tetrads. The colors from yellow to dark blue are arrangements of angle from low to high. The distance and angle criteria are 3.0Å and 135° respectively. The line colored in cyan represents the experimental value.

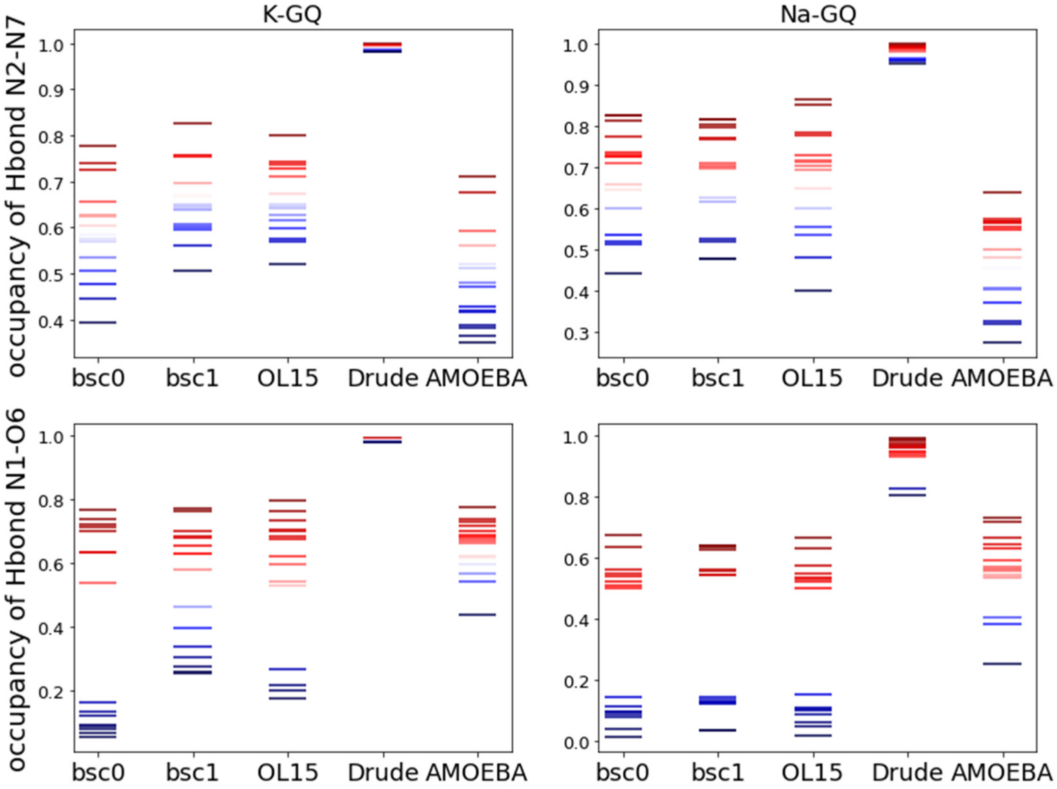

Figure S9. Occupancy of 32 hydrogen bonds during MD simulation. The colors from blue to red are arrangements of occupancy from low to high. The distance and angle criteria are 3.0Å and 135° respectively.

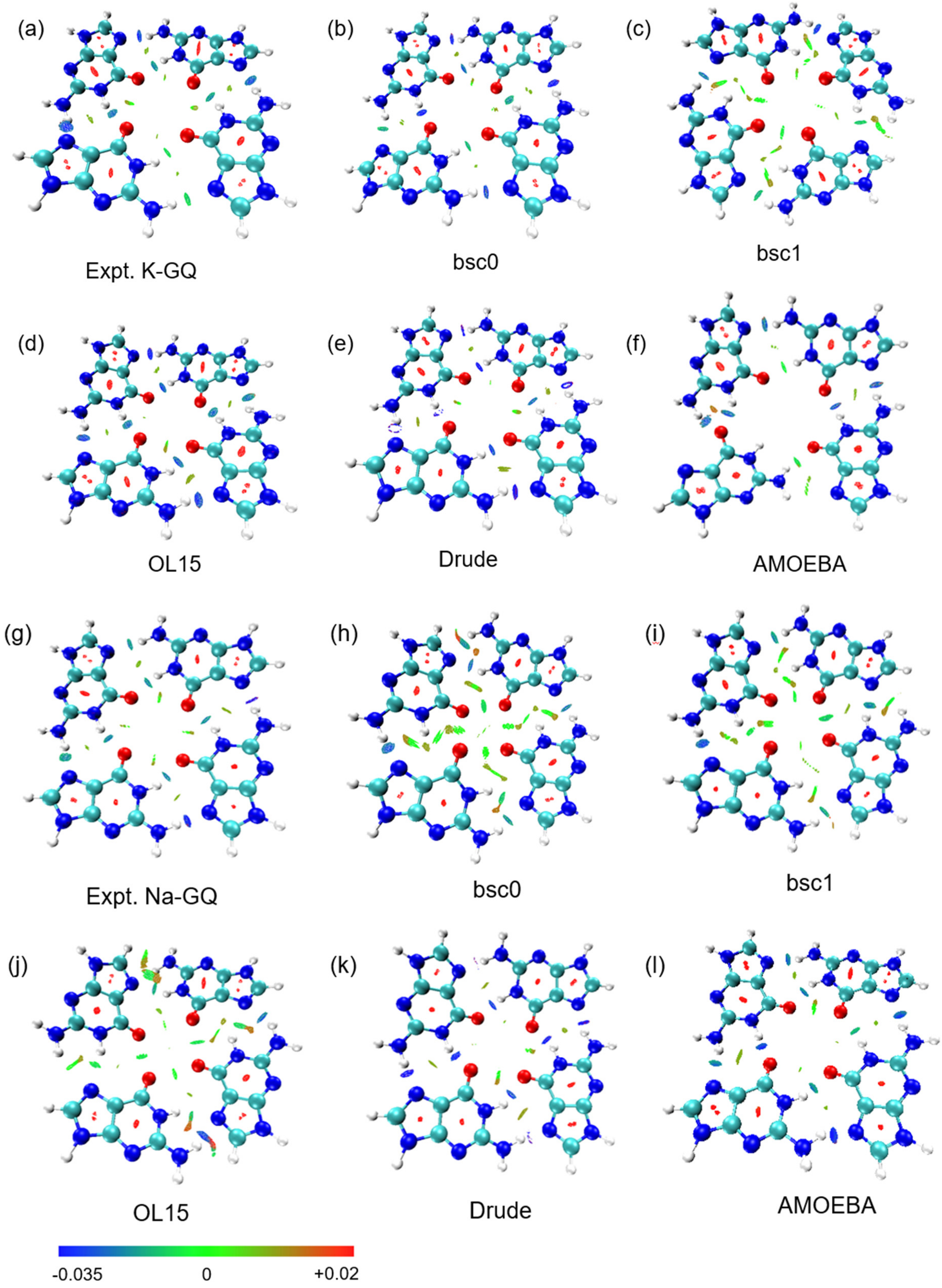

Figure S10. Hydrogen bonds interactions of G-quartets obtained from NCI (Non-Covalent Interaction) index calculation. The isosurface is 0.5 a.u. The pentose surgars were removed for clearity. (a)~(f) are quartets of K-GQ and (g)~(l) are quartets of Na-GQ.

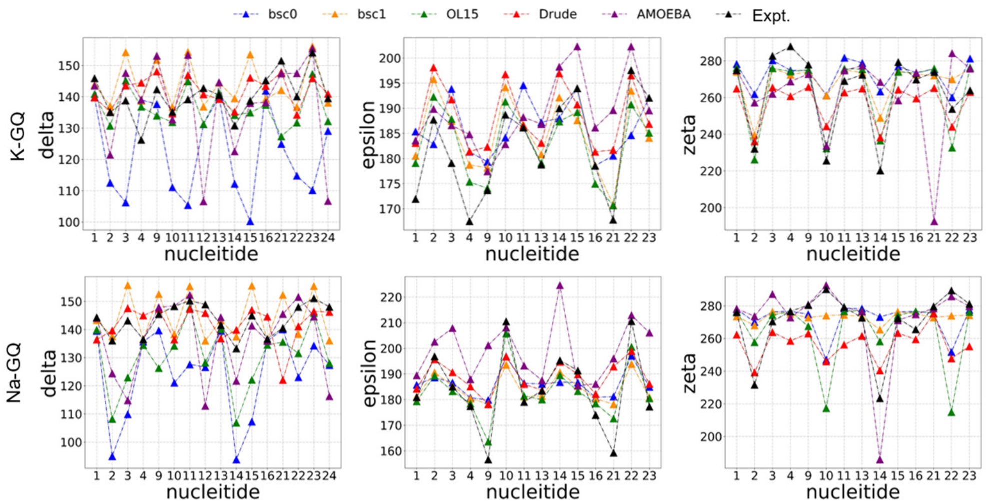

Figure S11. Average dihedral angle of guanine deoxyribonucleotides calculated from 2000 snapshots randomly extracted from the MD trajectories of K-GQ (upper panel) and Na-GQ (bottom panel), respectively. The experimental values are also shown for comparison.
